# On site discrimination between two closely related commercial strains of oyster mushroom using a loop-mediated isothermal amplification (LAMP) test

**DOI:** 10.1007/s11033-025-11156-0

**Published:** 2025-10-28

**Authors:** Johan Baars, Viola Kurm, Bart Scholten, Yvonne Griekspoor, Brian Lavrijssen, Jurre A. Steens, Marinus J. M. Smulders, Arend van Peer

**Affiliations:** 1 Plant Breeding, Wageningen University & Research, Wageningen, The Netherlands; 2 Biointeractions and Plant Health, Wageningen University & Research, Wageningen, The Netherlands; 3 Scope Biosciences BV, Wageningen, The Netherlands

**Keywords:** Oyster mushroom, Pleurotus ostreatus, LAMP, Plant breeder’s rights

## Abstract

**Background:**

Protection of the intellectual property (IP) rights on new crop varieties is important as it allows the breeding company or entity that produced the variety to earn back (part of) the investment. Infringement on the IP rights of mushroom varieties is not uncommon. In order to combat infringement of the IP rights on two strains of Oyster mushroom (SPOPPO and ALLERPO) it is important to be able to readily recognize and discriminate the two strains in commercial practice. This article describes the development of tools for the on-site identification of two closely related sporeless strains of Oyster mushroom.

**Methods and results:**

To develop a reliable method of discriminating between SPOPPO and ALLERPO, we used either the LAMP technique or a modification of that technique. It allows for fast (within 30 min) identification of the commercially used strains SPOPPO and ALLERPO with high specificity. Fast on-site answers on strain identity can be important when experiencing unexpected strain behavior or when strains are of suspect origin. Both strains are discriminated from sporulating strains by a LAMP reaction on the intact version of the *msh4* gene; sporeless strains contain a *msh4* gene with a large insert that renders the associated protein inactive.

**Conclusions:**

SPOPPO and ALLERPO are distinguished from each other and other commercially used *Pleurotus ostreatus* strains by LAMP reactions that target genomic regions with strain specific recombinations. To our knowledge, this is the first time LAMP reactions have been developed to discriminate between *Pleurotus ostreatus* strains.

**Supplementary Information:**

The online version contains supplementary material available at 10.1007/s11033-025-11156-0.

## Introduction

Oyster mushrooms (*Pleurotus* species) are among the most widely cultivated mushrooms in the world, together with *Lentinula edodes*, *Auricularia* species, *Agaricus bisporus* and *Flammulina* species [1]. In Europe, the main *Pleurotus* species that are being cultivated are King Oyster mushroom (*Pleurotus eryngii*) and Grey Oyster mushroom (*Pleurotus ostreatus*). Workers who are harvesting and handling the crop during the cultivation of Oyster mushrooms often develop respiratory problems. These are associated with the exposure to spores of *Pleurotus* spp [2–4]. Under controlled laboratory conditions it has been shown that the inhalation of spores of *P*. *ostreatus* provokes the development of an extrinsic allergic alveolitis [5].

Around the turn of the 20th century the Netherlands’ Ministry of Agriculture commissioned the development of a sporeless strain of Oyster mushroom to alleviate respiratory problems of workers in the cultivation of Oyster mushroom. This resulted in two sporeless varieties of *P. ostreatus* that are currently commercially available: SPOPPO and ALLERPO. Both the SPOPPO variety and the ALLERPO variety were developed in a breeding program based on crosses between a spontaneous sporeless *P. ostreatus* mutant, ATCC58937 (F42 × 11; [6]) and the commercial variety HK35. More detailed research has shown that the *poMSH4* gene in the sporeless *P*. *ostreatus* was interrupted by a large DNA fragment containing a region encoding a CxC5/CxC6 cysteine cluster associated with Copia-type retrotransposons [7]. A disruption of the homologous gene was shown to cause sporelessness in *Pleurotus pulmonarius* [8].

Many countries facilitate the protection of intellectual property rights on mushroom varieties that have been developed for horticultural purposes. Three systems can be discerned: plant breeder’s rights (PBR, also called plant variety protection or PVP) system, the Utility Patent, and (in the US) the Plant Patent [9]. The plant breeder’s right system is a dedicated system for plant and mushroom varieties based on the International Convention for the Protection of New Varieties of Plants. It is administered by the International Union for the Protection of New Varieties of Plants (UPOV), an intergovernmental organization with headquarters in Geneva (Switzerland). UPOV has developed Test Guidelines to discriminate between varieties based on phenotypic differences. The Guidelines detail the procedures for conducting tests, including the quantity and quality of plant material required, the duration of the tests and the conditions under which tests should be conducted. Use of the Test Guidelines ensures harmonized testing across different jurisdictions. For Oyster mushrooms, Test Guideline 291 applies (https://www.upov.int/edocs/tgdocs/en/tg291.pdf). Plant breeder’s rights can be granted, provided a variety is sufficiently distinct from existing varieties, uniform in its phenotypic characteristics and shows phenotypic stability over time. After having been granted plant breeder’s rights, the breeder has the exclusive rights to give licenses to others to multiply or use the variety and to charge a license fee on the multiplications or use of the variety for agricultural production (for a period of 20 or 25 years). A plant variety that is traded must be identified by its official name. This helps the owner to trace the use of its variety. The US has a similar type of protection for new varieties of non-tuberous, asexually propagated species, termed a “Plant Patent (PP)”. A Utility Patent is for inventions that are novel, inventive and industrially applicable [10]. It may cover plant parts or a plant with an inserted gene rather than a variety containing the gene [9]. Currently there are 15 Plant Patents known for strains of edible mushrooms [9, 11] and 27 US patents granted for new mushroom strains. Intellectual property rights of at least 45 varieties of *P. ostreatus* are protected (Supplementary file, Table 1). The varieties SPOPPO and ALLERPO are protected in the European Union, the United Kingdom and Israel by PBR and in the US by a Plant Patent.

Protection of the intellectual property (IP) rights on new crop varieties are important as it allows the breeding company or entity that produced the variety to earn back (part of) the investment. For breeding programs that are not publicly funded, IP protection is generally considered to be indispensable [11]. Without protection of IP rights, hardly any new mushroom strains would enter the market, and as a result the industry would not develop as well as it potentially could or even diminish when existing strains no longer suffice contemporary needs.

Infringement on the IP rights of mushroom varieties is not uncommon. As fungi are vegetatively propagated, it is very easy to make copies of varieties through tissue cultures. A well-known case within the mushroom industry is the infringement of the IP rights on strains from spawn company Amycel [9, 12, 13]. Also in case of *P. ostreatus* variety SPOPPO, incidents of infringement of the IP rights have occurred. In order to combat infringement of the IP rights on SPOPPO and ALLERPO, it is important to be able to readily recognize and discriminate the two strains in commercial practice. Both strains do not produce basidiospores and as such distinguish themselves from all other commercial strains currently on the market (with the exception of the ‘Purati’, ‘Karabella’ and ‘SPX’ that are also sporeless but have pronounced morphological differences). When seen side by side in the same growing room, the color difference between SPOPPO and ALLERPO is clear. However, when encountering one of the two strains by itself in a growing room, it is very difficult to determine the identity. As phenotypic tests conducted under the provisions of the UPOV Test Guideline are expensive and time-consuming, it would help if there is a first indication of a potential infringement when a suspect strain is located at a spawn company, substrate producer or mushroom farm. We therefore looked for a molecular method that may be used in the field to differentiate the two strains.

Loop-mediated isothermal amplification (LAMP) is a molecular technique [14] that is frequently used for the detection of plant pathogens. It makes use of up to six primers and a Bst DNA polymerase with strand displacement activity for amplification of nucleic acids at an isothermal temperature. A large amount of amplicon is produced that can be visualized by either end-point detection using, for example, intercalating dyes or ion indicators or by real-time detection, generally with intercalating fluorescent dyes [15]. Due to the number of primers LAMP is reported to be highly species-specific, allowing for the differentiation of various bacteria, fungal and viral pathogens [16–18]. In addition, combining LAMP pre-amplification with a CRISPR-detection step [19], allows for differentiation between targets on the basis of a single nucleotide polymorphism (SNP). Another advantage of LAMP is its potential for on-site detection, making it an ideal tool for distinguishing closely related organisms outside of a laboratory. The LAMP polymerase is less sensitive to inhibitors than commonly used PCR polymerases, and therefore crude, rapid extracts from various sources are often sufficient [20]. In addition, isothermal amplification and result read-out can be performed with portable equipment [21].

Due to its specificity, speed of operation and on-site applicability LAMP has been used previously for distinguishing plant species and even cultivars. A LAMP assay with end-point detection has been developed for the specific identification of saffron (*Crocus sativus*) that enabled its distinction from commonly used plants used to adulterate saffron [22]. LAMP assays have been used to identify transgenic rice based on the junction sequences of the transgenic events [23]. LAMP also makes it possible to distinguish strains of *Cannabis sativa* based on several single nucleotide polymorphisms (SNPs), indicating the high specificity of this technique [24]. In addition, Kitamura and colleagues [24] developed a simplified protocol for DNA extraction and amplicon visualization allowing for quick on-site detection. We are not aware of studies using LAMP for the discrimination of mushroom cultivars. We aim to base the assay both on DNA features close to the mutated gene and on strain specific recombination sites. A LAMP test close to the mutated gene would discriminate the sporeless strains SPOPPO and ALLERPO from all sporulating strains. For differentiation of SPOPPO and ALLERPO from each other we propose to use LAMP tests spanning specific recombination sites on the chromosomes. Genomic maps *of P. ostreatus* show a more or less even distribution of recombination events across linkage groups [25–27]. As the odds of finding specific recombinations are very low, the test should also be useful to differentiate the two sporeless strains from all other strains.

## Materials and methods

### Strains used

For the development and validation of the LAMP assays for the varieties SPOPPO and ALLERPO, additionally the sporeless strain ATCC58937 and the sporulating varieties HK35, P24, P80 and 3015 were used (Table 1). The fungal cultures were stored in liquid nitrogen and revived when needed. Mycelium was grown on malt extract agar. Spawn was either obtained from Sylvan Spawn (Langeais, France) or was made from sorghum grains. Preparation of spawn and colonized substrate was performed as described by Lavrijssen and colleagues [7]. Raw materials for preparing colonized substrate were kindly provided by VeMe Specials, Gemert, The Netherlands.

**Table 1:** Oyster mushroom strains used in this study

Strain	Sporulation	Commercial vendors
SPOPPO	No	Sylvan Spawn (Langeais, France)
ALLERPO	No	Amycel (Vendôme, France)
ATCC58937	No	No longer commercially available
HK35	Yes	Sylvan Spawn (Langeais)
P24	Yes	Italspawn (Onigo, Italy)
P80	Yes	Italspawn (Onigo, Italy)
3015	Yes	Amycel (Vendôme, France)

**Fig. 1:**
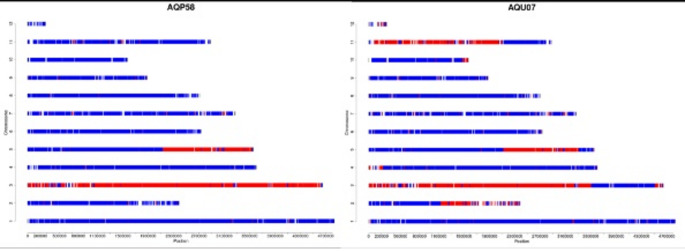
Location of recombination events in strains AQP-58 and AQU-07. Blue represents genetic material originating from strain HK-35, while red indicates genetic material originating from strain ATCC58937

The varieties SPOPPO and ALLERPO have been developed in a breeding program based on the strains HK35 and ATCC58937 as starting material [28, 29]. Strain ATCC58937 donated the sporeless trait. Figure 1 depicts the genomic make-up of two of the three monokaryotic strains that were used to make the varieties SPOPPO (cross of AQP58 and ASA53) and ALLERPO (cross of AQU07 and ASA53). The specific locations of the recombinations discriminate between these two varieties. Since both SPOPPO and ALLERPO contain genomic material of ASA53, only recombinations in the genomes of AQU07 and AQP58 are available to differentiate between SPOPPO and ALLERPO. Monokaryotic strain AQP58 only shows a recombination on linkage group 5 while AQU07 shows recombinations on chromosomes 2, 3, 4, 5, 10 and 11.

### Primer sequences

One primer set for identification of both sporeless varieties SPOPPO and ALLERPO was designed on the left flank of the msh4-gene insert present in the sporeless strains, partly extending over the insert (for a schematic depiction see Supplemental Fig. 1, for primer sequences see Table 2). The primer set AQU07 was designed to target a sequence on the AQU07 chromosome 3, since alignment of the respective sequences from AQP58, AQU07, HK35 showed differences in a few SNPs between AQU07 and AQP58 (Supplemental Fig. 2a). The primer set AQP58 was designed to target a sequence on the AQP58 chromosome 5 differing in a few SNPs from the corresponding sequence on AQU07, which were identified by an alignment of AQU07, AQP58 and HK35 as well (Supplemental Fig. 2b). Both the AQU07 and AQP58 primer sets also target the corresponding sequence on HK35. Therefore, the MSH4 assay is also required for strain identification. Alignments were done with CLC Genomics Workbench 24. All primers were designed using Primer Explorer v. 5.

The standard LAMP assay AQP58 was not sufficiently specific and was therefore used as a base for the design of a LAMP CRISPR-Cas (LAMP-CC) assay.

The addition of the CRISPR-Cas based read-out of the ScopeDx assay to the LAMP assay increases its specificity to enable the required distinction between AQP58 and AQU07. A crRNA is designed which together with Cmr subunits forms a CRISPR-Cas complex to distinguish between the LAMP-amplicons of AQP58 and AQU07 (Table 2). Only when perfect complementarity between crRNA and AQP58 is present, the CRISPR-Cas complex is activated which sets off a signaling cascade that generates a fluorescent probe-based read-out.

**Table 2:** Primer sequences targeting the msh4 insert, the ALLERPO specific AQU07 and the SPOPPO specific AQP58 and CrRNA sequence

Primer	Sequence 5’−3’	Target
MSH4-F3	AAATCGGTCACTGTCGGA	msh4 insertion
MSH4-B3	ACTGGATTCAGTTGGTGTT	msh4 insertion
MSH4-FIP	CTTTTTGAGTGCAAAAACAAACCCGGTGCACTCATTATCATTGTC	msh4 insertion
MSH4-BIP	ACTTCCAAGTGGCTTTATCAATGCCACTTGTAGACGTGGTGA	msh4 insertion
MSH4-LoopB	AGAGGAAGCTGTATATCTGGCAGT	msh4 insertion
AQU07-F3	AAGTTGGCACAGCCATGT	AQU07
AQU07-B3	CCGTTCCTTCTCCACAACAT	AQU07
AQU07-FIP	GCACGGGTTCCTGGAGCAATGTACCTCTGCTTTCCCCCA	AQU07
AQU07-BIP	CGAGGTGGCAGTAGCCTTGCCAAGCGCCTCAATTTTGACA	AQU07
AQU07-LoopB	CCGGCATGAGTGGAAAGGCA	AQU07
AQP58-F3	GGAAGGCCTTGAACACTGT	AQP58
AQP58-B3	TCGGGATGGCCTACATTGA	AQP58
AQP58-FIP	CCAGGGCACGAGTGTATAGTCGGCCAGGACGTTTCGCTTC	AQP58
AQP58-BIP	CAACCTTACCGCGATCCGTCAACAGCCAGGCATCTGTCA	AQP58
AQP58-LoopF	GTAGCATTCCGGAGGGGT	AQP58
AQP58-LoopB	CTCTCAACAGCTAGTTCAGCG	AQP58
crRNA	AUUGCGACGCUAGUUCAGCGCUGGAAUAUGACAGAUGCCUGGCUGU	

### LAMP assay

Primer mixes were prepared for the MSH4 and AQU07 primer sets consisting of 5 µM F3 and B3 primer, 20 µM FIP and BIP primers and 10 µM Loop primer. Each reaction mix contained 15 µl ISO-001 mix (OptiGene), 1 µl primer mix, 4 µl HyClone water (Cytiva) and 5 µl sample extract. The assays were performed at 65 °C for 40 min followed by a melting curve at 98 °C–65 °C.

The LAMP-CC method is based on a method developed by Steens et al. (2021) and further developed by Scope Bioscience to a one-pot assay. The reaction mix contained 15 µl master mix (Scope Biosciences) and 5 µl sample extract and the reaction was performed at 65 °C for 40 min.

Combination of the three LAMP assays allow discrimination between SPOPPO and ALLERPO. A positive signal in both the MSH4 and AQU07 LAMP assays is indicative for ALLERPO. A positive signal in both the AQP58 (LAMP-CC method) and the MSH4 LAMP assay is indicative for SPOPPO.

### Sample extraction

Mycelium extracts were made from Oyster mushroom mycelium grown on agar petri dishes by scraping off a small amount of mycelium using a 1 µl inoculation loop and transferring it to a 1.5 ml Eppendorf tube with 100 µl PEG lysis buffer (OptiGene). The mycelium was crushed manually using a miniature pestle for 60 s. The samples were incubated for 20 min at 95 °C and subsequently diluted 50 times in sample dilution buffer (OptiGene). Of these extracts 5 µl was used per LAMP reaction.

In addition to mycelium, all LAMP assays were performed on mushroom caps, spawn and colonized substrate (wheat straw based). For colonized substrate the LAMP assays were performed on substrate colonized by either SPOPPO, ALLERPO, HK35, P24, P80 and 3015.

Mushroom cap extracts were made using 125 mg mushroom tissue. The tissue was added to a 5 ml extraction tube containing 1 ml PEG lysis buffer (OptiGene) with 40 w/v % Chelex 100 and a metal ball (Ø 11.1 mm). After shaking the tube by hand for 1 min the samples were incubated for 20 min at room temperature and subsequently diluted 50 times in sample dilution buffer (OptiGene). Of these extracts 5 µl was used per LAMP reaction.

Oyster mushroom strains are commercially available as millet grains covered with mycelium. Spawn extracts were made using 2, 6 or 10 kernels in a 5 ml extraction tube with 1 ml PEG lysis buffer (OptiGene), 40 w/v % Chelex 100 and a metal ball (Ø 11.1 mm). The tubes were shaken for 1 min by hand followed by a 20-minute incubation at room temperature. Subsequently the extract was diluted 50 times with sample dilution buffer (OptiGene). Of these extracts 5 µl was used per LAMP reaction.

In Europe, Oyster mushroom strains are mostly grown on a substrate of pasteurized wheat straw. For extraction from colonized substrate 1–2 straws of substrate partly covered in mycelium were placed in a 5 ml extraction tube with 1 ml PEG lysis buffer (OptiGene), 40 w/v % Chelex 100 and a metal ball (Ø 11.1 mm). After shaking the tube by hand for 1 min and 5-minute incubation at room temperature the samples were diluted 50 times with sample dilution buffer (OptiGene). Of these extracts 5 µl was used per LAMP reaction.

### Specificity

For assessing the specificity of the three assays, all were tested on mycelium extracts of the non-sporulating strains SPOPPO, ALLERPO, and ATCC58937 and on the sporulating strain HK35. In addition, specificity was tested on mushroom caps of the non-sporulating strains SPOPPO and ALLERPO and the sporulating strains HK35, P24, P80 and 3015.

## Results

The three different LAMP assays were tested on a number of different sample types as they may appear in commercial practice.

### Specificity test on mycelium

**Fig. 2:**
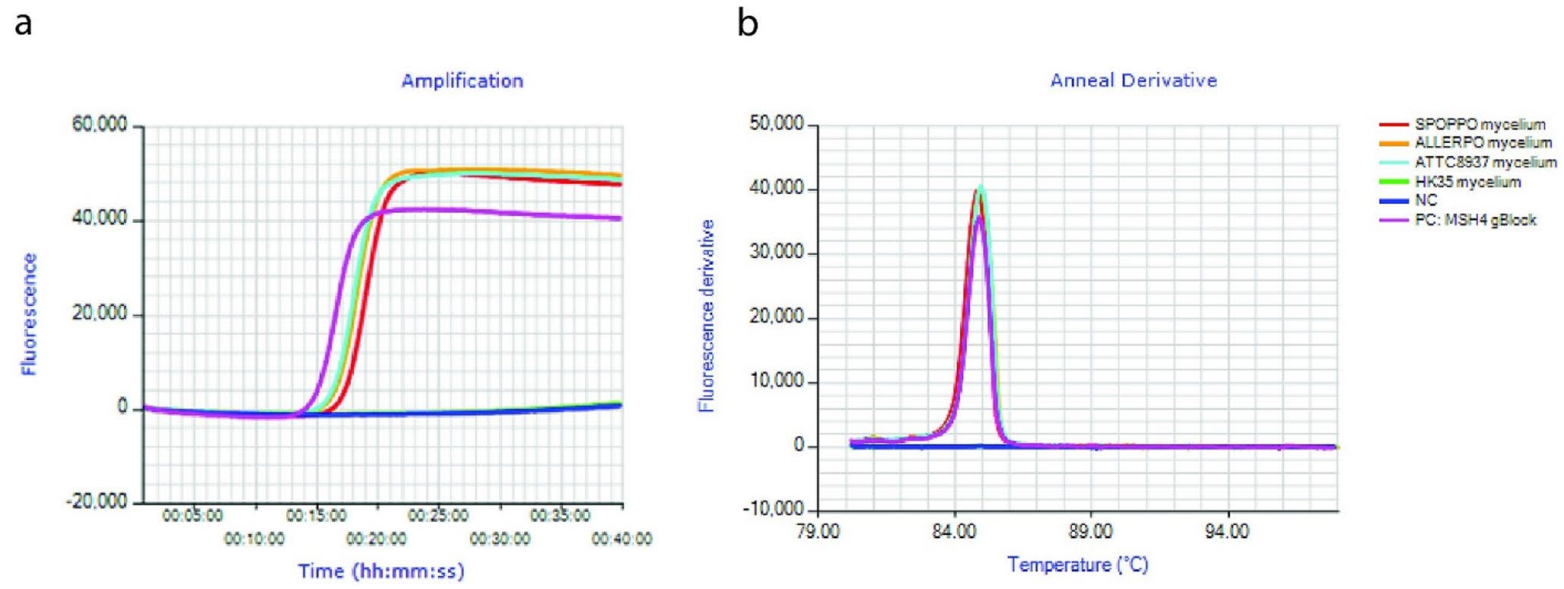
(**a**) Amplification curves and (**b**) melting curves of a msh4-LAMP assay on mycelium samples of SPOPPO, ALLERPO, ATCC58937 and HK35, a negative control, and a gBlock of the disrupted msh4-gene as a positive control

With the MSH4-assay, extracts from SPOPPO, ALLERPO and ATCC58937 mycelium showed a positive reaction with a time of positivity of 17:45 − 18:30 min and a melting temperature of approximately 84.9 °C (Table 3; Fig. 2). There was no amplification with the HK35 extract. The AQU07 assay showed positive amplification with the ALLERPO mycelium extract and the HK35 mycelium extract, but not the SPOPPO and ATCC58937 mycelium extracts (Table 3 Supplemental Fig. 3). Time of positivity for the ALLERPO target was 20:15 min with a melting temperature of 89.6 °C. The LAMP-CC AQP58 assay in turn amplified SPOPPO and HK35 mycelium, but not ALLERPO mycelium with an approximate time of positivity of 18:00–19:00 min (Table 3, Supplemental Fig. 4). Due to the use of a fluorescent probe, this assay does not produce a melting curve.

### Specificity test in a mushroom cap matrix

With mushroom caps, following a simple DNA extraction, the MSH4-assay showed a positive reaction with the sporeless varieties SPOPPO and ALLERPO, but not with the sporulating varieties 3015 (Amycel) and P24 and P80 (Italspawn) (Table 3). SPOPPO and ALLERPO could be distinguished with the AQU07 and AQP58 assays specific for ALLERPO and SPOPPO respectively. The latter two assays also showed positive reactions with sporulating varieties.

### Test in a spawn matrix

The MSH4 and the AQP58 assay, but not the AQU07 assay were positive for all SPOPPO spawn samples (Table 3, Supplemental Fig. 5–7). Two kernels of spawn were sufficient for a positive reaction within 24 min.

**Table 3:** Overview of time of positivity (Tpos) and melting temperature (Tm) of two LAMP and one LAMP-CC assay for the msh4-gene, ALLERPO and SPOPPO respectively on various types of sample; n.d.=not done, n.a. = not applicable

Experiment	Strain	Sample type	Target				
			msh4 insert		AQU07 (ALLERPO specific)		CRISPR-Cas AQP58 (SPOPPO specific)
			Tpos (min.)	Tm (°C)	Tpos (min.)	Tm (°C)	Tpos (min.)
Mycelium tests	SPOPPO	mycelium	18:30	84.8	-	-	18:15–19:00 (duplicates)
	ALLERPO	mycelium	18:00	84.9	20:15	89.6	-
	ATCC58937	mycelium	17:45	84.9	-	-	-
	HK35	mycelium	-	-	22:00	89.5	18:30 − 19:15 (duplicates)
	NC	water	-	-	-	-	-
	PC	msh4 gBlock	16:15	84.9	n.d.	n.d.	n.d.
	NC	AQU07 gBlock	n.d.	n.d.	n.d.	n.d.	-
	PC	AQP58 gBlock	n.d.	n.d.	n.d.	n.d.	15:45
Mushroom tests	SPOPPO	Mushroom 1	20:15	84.8	-	-	18:30
	SPOPPO	Mushroom 2	20:30	89.4	-	-	18:00
	ALLERPO	Mushroom 1	20:00	85.0	25:15	89.4	-
	ALLERPO	Mushroom 2	19:45	85.0	25:30	89.4	-
	3015 Amycel	Mushroom	-	-	23:00	89.5	19:00
	P24 Italspawn	Mushroom	-	-	24:00	89.4	18:45
	P80 Italspawn	Mushroom	-	-	-	-	18:30
	NC	AQU07 gBlock	n.d.	n.d.	n.d.	n.d.	-
	PC	AQP58 gBlock	n.d.	n.d.	n.d.	n.d.	16.30
	PC ALLERPO	mycelium	19:45	84.9	25:45	89.5	n.d.
	PC HK35	mycelium	-	66.7	26.15	89.5	n.d.
	PC SPOPPO	mycelium	n.d.	n.d.	-	-	n.d.
	NC	water	-	-	-	-	-
Spawn tests	SPOPPO	Spawn; 2 kernels	24:00	84.6	-	-	21:30
	SPOPPO	Spawn; 6 kernels	22.45	84.6	-	-	20:30
	SPOPPO	Spawn; 10 kernels	22.00	84.6	-	-	20:00
	NC	AQU07 gBlock	n.d.	n.d.	n.d.	n.d.	-
	PC	AQP58 gBlock	n.d.	n.d.	n.d.	n.d.	17:45
	PC ALLERPO	mycelium	n.d.	n.d.	22:30	89.5	n.d.
	PC SPOPPO	mycelium	20:30	84.9	n.d.	n.d.	n.d.
	NC	water	-	-	-	-	-
Substrate tests	SPOPPO	Substrate	18:30	84.7	-	-	16:45
	ALLERPO	Substrate	18:45	84.7	20:45	89.5	-
	P80	Substrate	-	-	-	-	16:45
	HK35	Substrate	-	-	21:30	89.4	18:15
	PC SPOPPO	mycelium	n.d.	n.d.	n.d.	n.d.	18:30
	PC ALLERPO	mycelium	24:00	84.8	22.45	89.5	n.d.
	NC	water	-	-	-	-	-

With respect to the CRISPR-CAS AQP58 assay; due to the use of a fluorescent probe, this assay does not produce a melting curve

### Test in substrate

The three LAMP assays specifically identified SPOPPO and ALLERPO in colonized wheat straw based substrate. The MSH4 assay showed positive amplification for ALLERPO- and SPOPPO-colonized substrate, but not for substrate colonized by P80 and HK35 (Table 3, Supplemental Fig. 8–10). The AQU07 assay was positive with ALLERPO-colonized substrate (Supplemental Fig. 9) and the AQP58 assay was positive with SPOPPO-colonized substrate (Supplemental Fig. 10). Time of positivity for the different assays was between 16 and 21 min.

## Discussions and conclusions

The unambiguous identification of mushroom varieties in an on-site setting is of high importance for the protection of IP rights. In many countries, infringement of PBR/PVP is punishable by law [30]. On the one hand infringement of PBR/PVP is hard to prove as evidenced by the case of lettuce seed [31]. On the other hand, one should be cautious not to falsely accuse people or organizations. Knowing early on that infringement is a possibility on the basis of on-site identification of strains, may help building a legal case. Such a method may also serve as a way for spawn companies to verify if growers have used their strains in case cultivation problems have risen that are accompanied by deviant mushroom morphology or even lack of mushrooms.

Here we have developed three LAMP-(based) assays that together can identify the varieties SPOPPO and ALLERPO and distinguish them from all tested sporulating strains and the sporeless parent ATCC58937. The MSH4 LAMP assay targets the region of an insertion in the *msh4* gene, which is responsible for the non-sporulating phenotype. Our MSH4 assay amplified varieties with this particular insertion with a high specificity, as no other varieties without this insert showed a reaction with the MSH4 assay. It is unlikely that new oyster mushroom varieties developed from other sporeless parents than ATCC58937 will contain the exact same insertion. Therefore, the MSH4 LAMP assay is expected to be suitable for the specific identification of SPOPPO and ALLERPO.

Developing specific LAMPs to distinguish the two sporeless varieties SPOPPO and ALLERPO was more challenging since both share one parent haplotype (ASA53) while the complementing differing parent haplotypes (AQU07 or AQP58) are closely related. Nevertheless, the developed AQU07 assay showed a positive reaction with ALLERPO, but not with SPOPPO. This specificity is likely based on several SNPs occurring in the primer binding region near the 3’ end of the FIP (F1c + F2) primer as the primers’ 3’ end is highly important for annealing and elongation [32]. However, it was not possible to design a LAMP assay specific for AQP58 present in SPOPPO. Therefore, a LAMP-CC assay [19] was needed to distinguish SPOPPO from ALLERPO with a positive reaction. Addition of the AQP58-specific CRISPR-Cas complex enabled binary distinction between SPOPPO and ALLERPO. This illustrates the added value of the CRISPR-Cas based detection, when only minimal genetic differences exist, without increasing detection time. Both assays showed amplification with the parent HK35 and other sporulating varieties. Therefore, the MSH4 assay is required in addition for specific identification.

In this study we tested if the three developed assays can be used with three different matrices, namely mushroom fruiting bodies, spawn and colonized substrate. Spawn is typically produced with millet, sorghum or wheat grain, while substrate contains straw. These materials often contain inhibitors such as humic acids and polysaccharides. However, all three assays (MSH4, AQU07 and AQP58) showed amplification as expected in all three tested materials with no increase in time of positivity. Thus, a simple extraction procedure was sufficient for strain identification in all substrates. Therefore, the LAMP assays are suitable for on-site application. In addition, we demonstrated that already two kernels of spawn or one piece of colonized straw substrate is sufficient for a positive reaction.

The validation of detection assays typically involves testing of sensitivity and of inclusivity. However, sensitivity is not of concern for these assays since they are intended to be used for identification in cases of abundant available material. Inclusivity is also not applicable in this case since there are only two varieties that were targeted by the developed assays.

On-site applicability of assays for identity confirmation in cases of suspected IP infringements is crucial. Current molecular techniques for strain identification require laboratory equipment and trained personnel for DNA extraction and subsequently conducting the respective tests. Moreover, the transportation of samples from the breeder to the laboratory requires a chain of procedures to be followed to ensure that the samples are not compromised. In addition, in the time from sampling until obtaining the results, material with potentially copied mushroom strains can be destroyed or removed. Therefore, a LAMP assay that can be performed on-site, and works with various matrices, is highly advantageous. Similarly, when problems in production are encountered, a rapid confirmation of strain identity is desirable.

Still, a few challenges remain for the use of LAMP in general and the assays developed here specifically. While performing a LAMP assay is relatively simple compared to other molecular techniques, it still requires basic laboratory skills such as pipetting and consumables, such as primers, enzymes and specific tubes that are relatively costly. In addition, the read-out method in the case of this study relies on real-time fluorescence detection, for which a (portable) device such as a Genie III (OptiGene) is needed. Alternative read-out methods include end-point read-outs relying on a color change or an increase in turbidity, which do not require a read-out device. However, these methods do not allow for assessing the melting temperature and therefore can lead to false positives. In addition, there is of yet no alternative read-out method for the LAMP-CC assay, which relies on a fluorophore signal. Currently, smaller and more cost-efficient devices have been developed, for instance the Genie-Lite system from OptiGene (https://www.optigene.co.uk/instruments/genie-lite/). However, these remain to be implemented in practice.

## Supplementary Information

Below is the link to the electronic supplementary material.

Supplementary Material 1

## Data Availability

No datasets were generated or analysed during the current study.
