# Supplementary material for: On site discrimination between two closely related commercial strains of oyster mushroom using a loop-mediated isothermal amplification (LAMP) test
Source: Mol Biol Rep. 2025 Oct 28;53(1):17. doi: 10.1007/s11033-025-11156-0 (PMC12568894; doi:10.1007/s11033-025-11156-0)
Supplement: Supplementary file 1 — Supplementary Material 1 [file 11033_2025_11156_MOESM1_ESM.docx]

On site discrimination between two closely related commercial strains of Oyster mushroom using a loop-mediated isothermal amplification (LAMP) test.

Johan Baars^a^, Viola Kurm^b^, Bart Scholten^c^, Yvonne Griekspoor^b^, Brian Lavrijssen^a^, Jurre A. Steens^c^, Marinus J.M. Smulders^a^, Arend van Peer^a^

^a^ Plant Breeding, Wageningen University & Research, Wageningen, The Netherlands

^b^ Biointeractions and Plant Health, Wageningen University & Research, Wageningen, The Netherlands

^c^ Scope Biosciences BV, Wageningen, The Netherlands

Corresponding author: Johan.Baars@wur.nl

Johan Baars (<https://orcid.org/0000-0002-0692-7371>), Viola Kurm (<https://orcid.org/0000-0003-1261-9778>), Bart Scholten (<https://orcid.org/0009-0003-4182-5455>), Yvonne Griekspoor (<https://orcid.org/0009-0004-6520-5733>), Brian Lavrijssen (<https://orcid.org/0000-0001-9309-1121>), Jurre A. Steens (<https://orcid.org/0000-0001-9293-7228>), Marinus J.M. Smulders (<https://orcid.org/0000-0002-8549-6046>), Arend van Peer (<https://orcid.org/0000-0001-7023-0045>)

Author Contributions

Conceptualization: Johan Baars, Viola Kurm, Jurre A. Steens, Marinus J.M. Smulders, Arend van Peer; Methodology: Viola Kurm, Bart Scholten, Yvonne Griekspoor, Brian Lavrijssen; Formal analysis and investigation: Viola Kurm, Bart Scholten , Yvonne Griekspoor, Brian Lavrijssen; Writing - original draft preparation: Johan Baars, Viola Kurm; Writing - review and editing: Johan Baars, Viola Kurm, Bart Scholten, Yvonne Griekspoor, Brian Lavrijssen, Jurre A. Steens, Marinus J.M. Smulders, Arend van Peer

**Supplemental data**

Supplementary Table 1. Overview of Pleurotus ostreatus varieties that are protected by either plant breeder's rights (PBR) or a plant patent (PP).

| Denomination | Geographic region | IP system | Registration no. | Expiration date |
| --- | --- | --- | --- | --- |
| SPOPPO | European Union | PBR | 18138 | 31/12/2031 |
|  | USA | PP | PP18037 | 07/07/2025 |
|  | UK | PBR | 27989 | 17/07/2031 |
|  | Israel | PBR | 3209 | 03/03/2037 |
| ALLERPO | European Union | PBR | 18139 | 31/12/2031 |
|  | USA | PP | PP32864 | 22/10/2039 |
|  | UK | PBR | 27990 | 17/07/2031 |
| Y6 | European Union | PBR | 55073 | 31/12/2045 |
| SY8 | European Union | PBR | 56398 | 31/12/2045 |
| TPLO1 | European Union | PBR | 55070 | 31/12/2045 |
|  | UK | PBR | 27988 | 20/04/2045 |
| TPLO4 | European Union | PBR | 55072 | 31/12/2045 |
|  | UK | PBR | 27987 | 20/04/2045 |
| TPLO5 | European Union | PBR | 55071 | 31/12/2045 |
|  | UK | PBR | 27986 | 20/04/2045 |
| HELIOS | European Union | PBR | 31888 | 31/12/2037 |
|  | UK | PBR | 27985 | 19/03/2037 |
| Ikaros | European Union | PBR | 31887 | 31/12/2037 |
|  | UK | PBR | 27984 | 19/03/2037 |
| Fotios | European Union | PBR | 31886 | 31/12/2037 |
|  | UK | PBR | 27983 | 19/03/2037 |
| HOX 1GO | European Union | PBR | 47440 | 31/12/2042 |
|  | Japan | PBR | 23540 | 08/09/2039 |
| Aran | South Korea | PBR | 9206 | 26/09/2042 |
| Baekseon | South Korea | PBR | 8017 | 03/03/2020 |
| Chunhwashim | South Korea | PBR | 6385 | 22/12/2036 |
| Daejang 2ho | South Korea | PBR | 5784 | 25/11/2035 |
| Daejang 3ho | South Korea | PBR | 5785 | 25/11/2035 |
| Dagul | South Korea | PBR | 6383 | 22/12/2036 |
| Dahyun | South Korea | PBR | 8467 | 08/03/2041 |
| Gonji 5ho | South Korea | PBR | 4374 | 11/03/2033 |
| Gonji 7ho | South Korea | PBR | 4751 | 05/01/2034 |
| Gosol | South Korea | PBR | 7520 | 06/02/2039 |
| Hantari | South Korea | PBR | 8464 | 08/03/2041 |
| Heuksol | South Korea | PBR | 7517 | 06/02/2039 |
| Heuksung | South Korea | PBR | 7521 | 06/02/2039 |
| Hwaseong 6ho | South Korea | PBR | 7453 | 01/01/2039 |
| Hwaseong 7ho | South Korea | PBR | 8370 | 13/12/2040 |
| Hwaseong 8ho | South Korea | PBR | 8488 | 30/03/2041 |
| Hwaseong 9ho | South Korea | PBR | 9200 | 12/09/2042 |
| Hwaseong 10ho | South Korea | PBR | 9292 | 09/01/2043 |
| Mongdol | South Korea | PBR | 5242 | 19/01/2035 |
| Sena | South Korea | PBR | 9180 | 16/08/2042 |
| Soltari | South Korea | PBR | 7397 | 25/10/2038 |
| Songnee | South Korea | PBR | 5249 | 19/01/2035 |
| Sootari | South Korea | PBR | 7985 | 13/02/2040 |
| Yasan | South Korea | PBR | 6384 | 22/12/2036 |
| Youngsan | South Korea | PBR | 8728 | 27/09/2041 |
| 3015 M | Russian Federation | ? | 73499 | ? |
| FUNGISEM K-15 | Russian Federation | ? | 42122 | ? |
| MOSKOVSKAYa | Russian Federation | ? | 49710 | ? |
| NK 35 | Russian Federation | ? | 31682 | ? |
| P 77 | Russian Federation | ? | 78500 | ? |
| P 80 | Russian Federation | ? | 78499 | ? |
| R 17 | Russian Federation | ? | 69499 | ? |
| SANTANA RF 336 | Russian Federation | ? | 42382 | ? |
| Silvan HK-35 | Ukraine | ? | 05274 | ? |


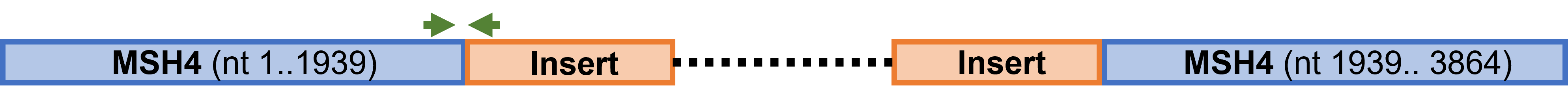


Supplemental Figure 1. Schematic depiction of the location of the LAMP assay for the identification of the insertion in the msh4 gene.

a)


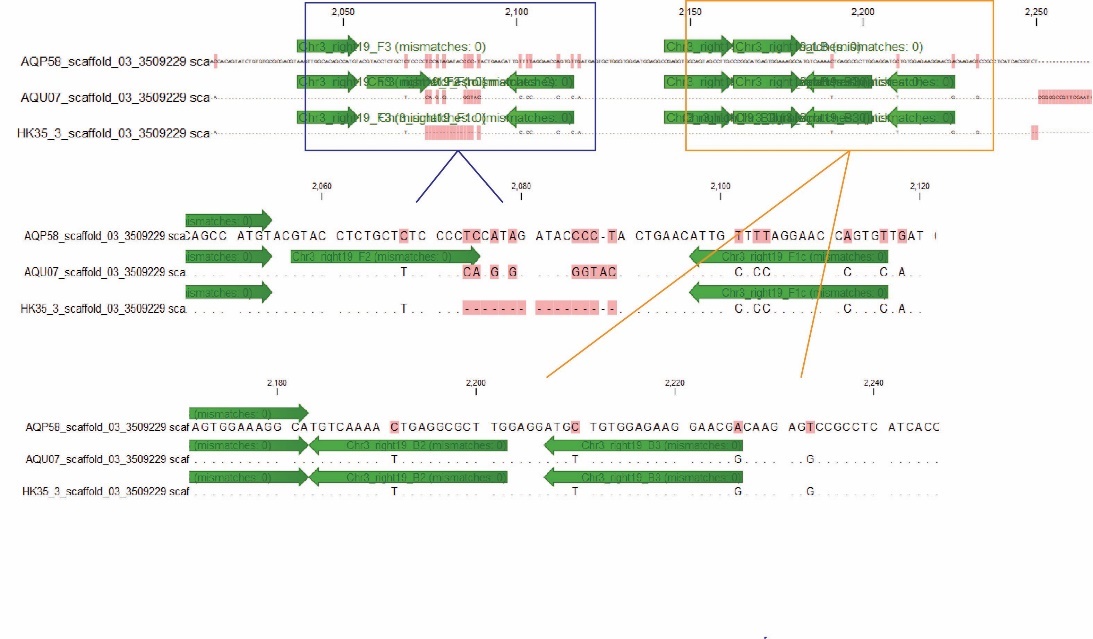


b)


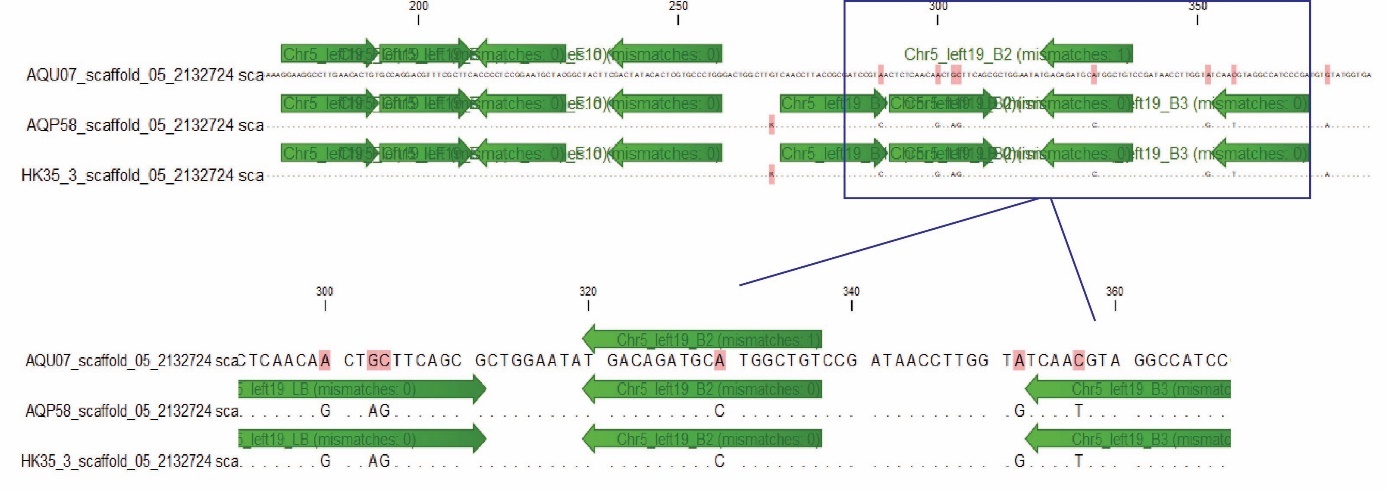


Supplemental Figure 2. Primer bindign sites of the primers of the LAMP assay for a) AQU07 on AQU07, AQP58 and HK35, and b) AQP58 on AQU07, AQP58 and HK35, including overview and focus on SNP regions.


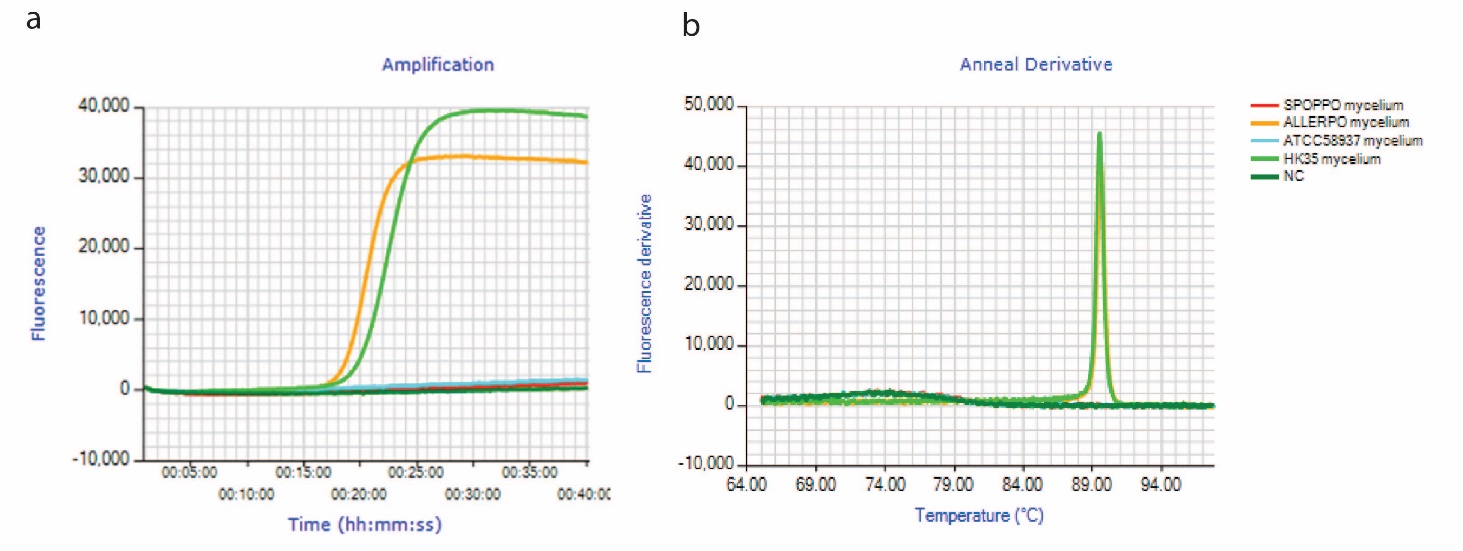


Supplemental Figure 3. a) Amplification curves and b) melting curves of a LAMP assay specific for AQU07 on mycelium of SPOPPO, ALLERPO, ATCC58937 and HK35 and a negative control.


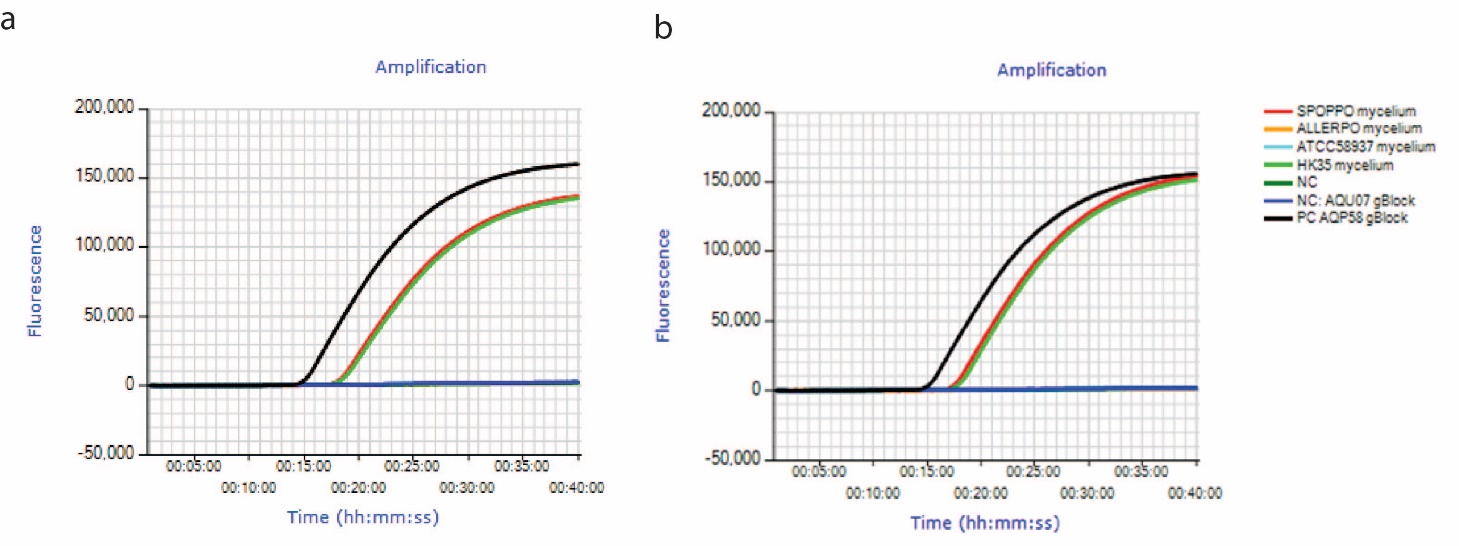


Supplemental Figure 4. Amplification curves of two replicates of a LAMP-CC assay specific for AQP58 on mycelium of SPOPPO, ALLERPO, ATCC58937 and HK35, a negative control, a AQP58 gBlock as a positive control, and the corresponding sequence on AQU07 as a gBlock as a non-template control; a) replicate 1, b) replicate 2.


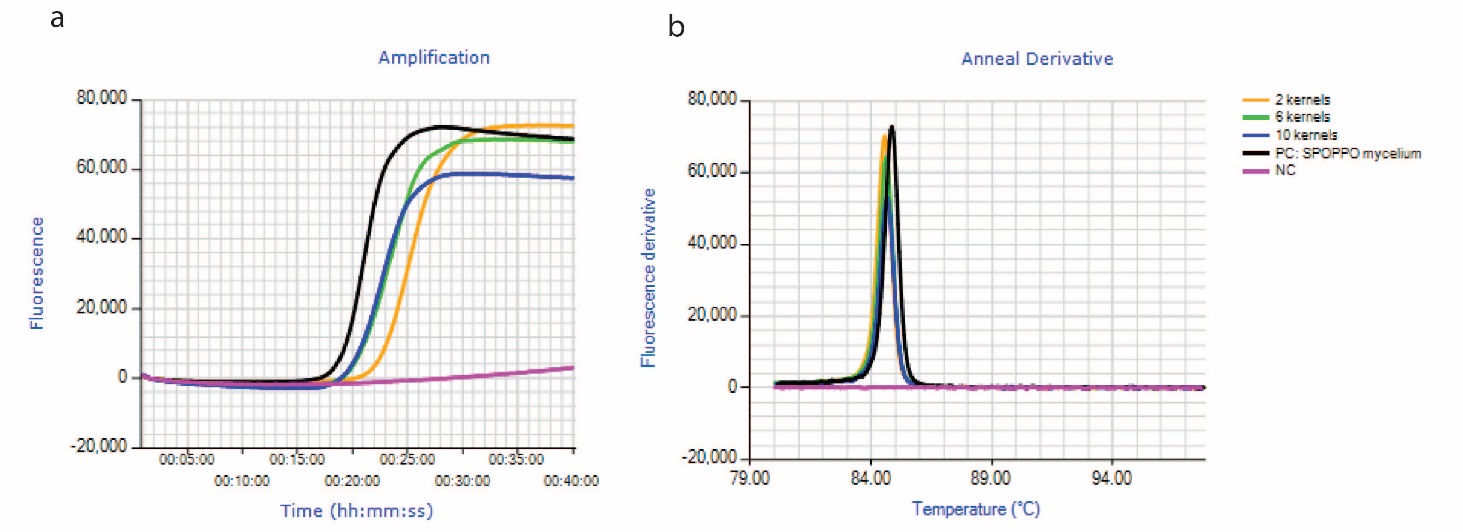


Supplemental Figure 5. a) Amplification curves and b) melting curves of a LAMP assay specific for the msh4-gene on kernels of spawn and SPOPPO-mycelium as a positive control.


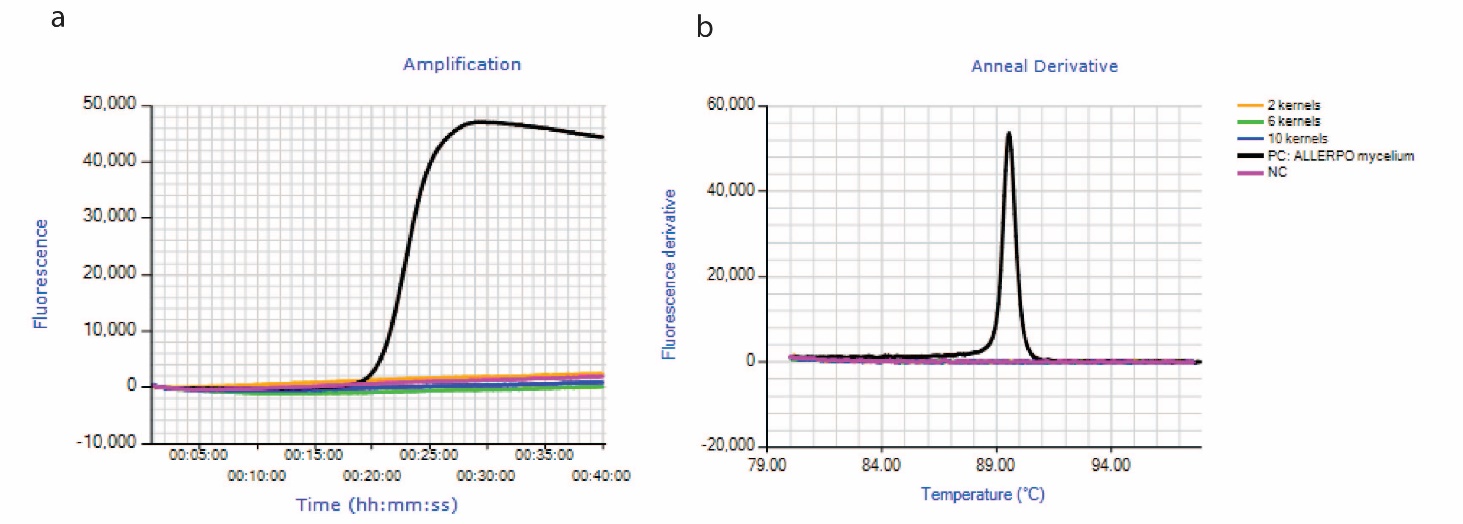


Supplemental Figure 6. a) Amplification curves and b) melting curves of a LAMP assay specific for AQU07 on kernels of spawn and ALLERPO-mycelium as a positive control.


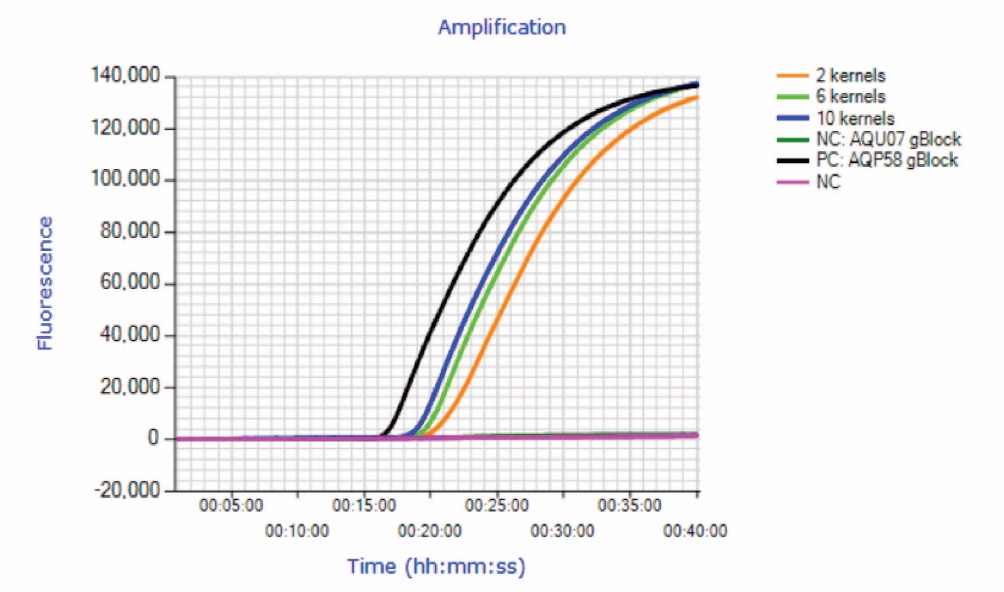


Supplemental Figure 7. Amplification curves of a LAMP-CC assay specific for AQP58 on kernels of spawn and the AQU07 gBlock as a non-template control and the AQP58 gBlock as a positive control.


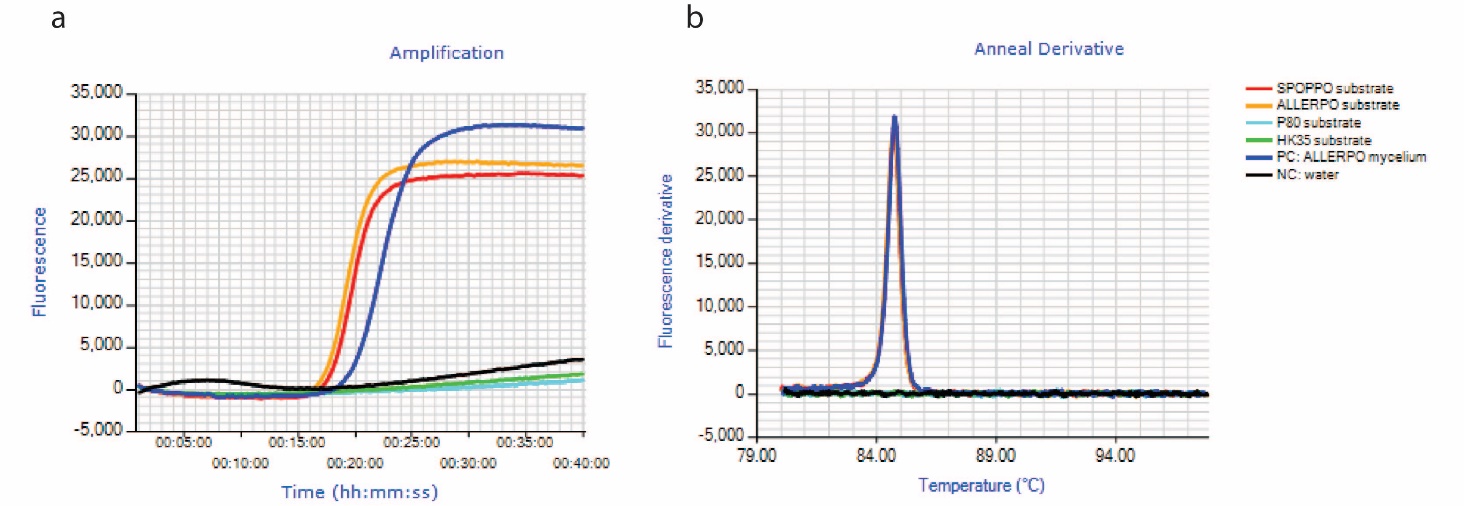


Supplemental Figure 8. a) Amplification curves and b) Melting curves of a MSH4-LAMP assay on substrate colonized by SPOPPO, ALLERPO, P80 and HK35, ALLERPO mycelium extract was used as a positive control.


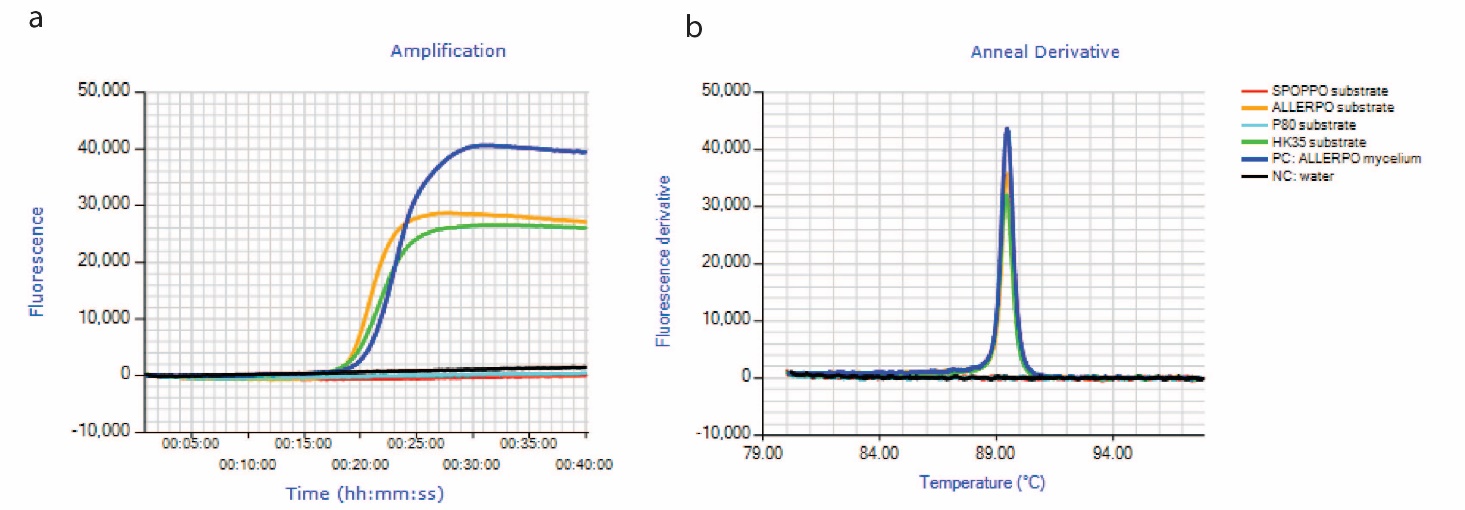


Supplemental Figure 9. a) Amplification curves and b) Melting curves of an AQU07 LAMP assay on substrate colonized by SPOPPO, ALLERPO, P80 and HK35, ALLERPO mycelium extract was used as a positive control.


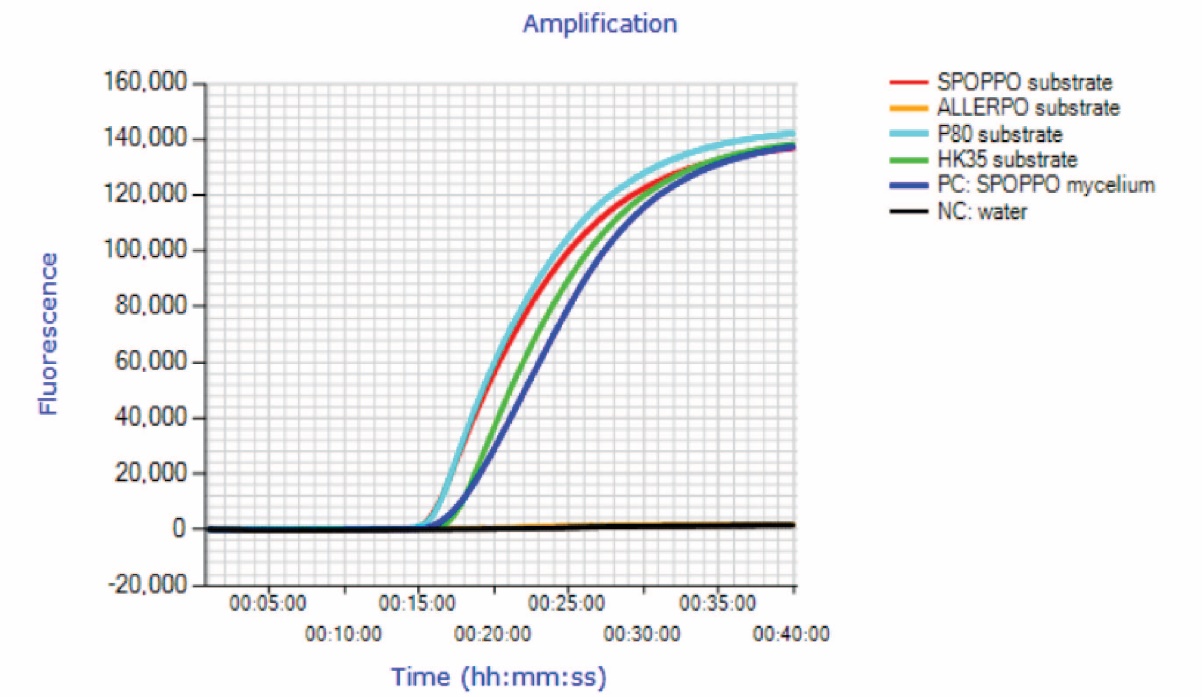


Supplemental Figure 10. Amplification curves of a AQP58 LAMP-CC assay specific for SPOPPO on substrate colonized by SPOPPO, ALLERPO, P80 and HK35, SPOPPO mycelium extract was used as a positive control.
